# Editorial: Costs and tradeoffs in plant adaptation and acclimation to metals

**DOI:** 10.3389/fpls.2023.1245426

**Published:** 2023-07-24

**Authors:** Ewa Muszyńska, Sabina Rossini-Oliva, Alina Wiszniewska

**Affiliations:** ^1^ Department of Botany, Institute of Biology, Warsaw University of Life Sciences-SGGW, Warsaw, Poland; ^2^ Department of Plant Biology and Ecology, University of Seville, Seville, Spain; ^3^ Department of Botany, Physiology and Plant Protection, University of Agriculture in Kraków, Kraków, Poland

**Keywords:** antioxidants, cysteine, metals, oxidative stress, photosynthesis, silicon, quercetin

The sessile lifestyle of plants forces them to confront dynamic changes in the surrounding environment which provoke a variety of structural, physiological, and genetic reactions leading either to disturbances in plant growth and even its dead or to the acclimation and adaptation to stressful conditions. An example of abiotic stressors, which strongly modify many critical parameters for plant survival, are metallic trace elements. Their enhanced release into the environment results mainly from human activities, such as smelting, mining, fertilizer use, the tanning industry, or waste incineration (Aslam et al.; El-Okkiah et al.; Kim et al.; Tan et al.). Nowadays, efforts are made to understand metal tolerance mechanisms functioning in unique plants, called metallophytes, that exhibit a higher toxicity threshold or even slightly beneficial effects of metallic ions on their growth in comparison with the representatives of non-metalliferous populations (Muszyńska et al., 2021; Rossini-Oliva et al., 2021). On the other hand, experiments are also focused on the search for chemical and physical methods of the alleviation of metal stress nuisance for non-metal tolerant species (Wiszniewska, 2021). Our Research Topic ‘*Cost and tradeoffs in plant adaptation and acclimation to metals*’ fits well with these novel approaches since it demonstrates the role of exogenous compounds in metal uptake and tolerance.

Kim et al. analyzed the effect of the exogenous application of 50 µM cysteine (Cys) on mercury (Hg) uptake and transport in *Arabidopsis thaliana* Col-0 plants. This amino acid enhanced Hg accumulation in the seedlings, but did not promote its translocation to the shoots. Hg was accumulated mostly in the roots (over 100-fold higher concentration than the shoots), and the uptake was facilitated by the formation Cys-Hg complexes, entering the roots most likely *via* the AtLHT1 transporter. Expression of genes encoding metal chelators (glutathione, phytochelatins, metallothioneins) was not affected by Cys in the case of seedlings, but in the case of roots and shoots several differential expression profiles were noted for glutathione synthases and metallothioneins (MT2 and MT3). Together with stimulation of metal chelator synthesis, exogenous Cys also boosted antioxidant response in Hg-treated *Arabidopsis*. There is evidence that Cys was directly involved in radical scavenging, but also modulated transcript levels of enzymatic antioxidants. Authors concluded that Cys increases Hg tolerance in *Arabidopsis*, although it also enhances metal accumulation in the roots. This finding, as well as an insight into the mechanism of Hg phytostabilization, may facilitate further studies on phytoremediation.

The role of quercetin (Qu) in ameliorating chromium (Cr) toxicity was evaluated by Aslam et al. in fenugreek (*Trigonella corniculata*). The seeds were primed with various doses of Qu prior to Cr treatment. Cr toxicity in non-primed plants was manifested by reduced biomass accretion, lower photosynthetic efficiency, and significant oxidative stress. The study revealed that a concentration of 25 µM Qu significantly improved growth parameters, as well as several biochemical characteristics, such as protein, fiber, and flavonoid content, proline accumulation and concentration of photosynthetic pigments. Interestingly, plants benefited from Qu application also in non-stressful conditions, without exposure to Cr. In the presence of Cr, the most pronounced effect of Qu priming was the elevated activity of antioxidant enzymes. In conclusion, the Authors proposed to test the Qu effect also for other abiotic stresses, as this is a non-harmful compound that may accelerate plant recovery in stressful conditions.

Another approach to mitigate metal toxicity was tested by El-Okkiah et al. in pea (*Pisum sativum*). They employed foliar application of silicon (Si) to improve plant growth in the soil contaminated with cadmium (Cd), and to evaluate its effects on anatomical and biochemical features. The authors conducted data modelling with the use of an artificial neural network to get an insight into the mechanisms of Cd-Si interactions. They revealed that Si ameliorated Cd-induced disturbance in organ anatomy, increasing lignification and other modifications of the cell walls due to deposition of Si inside root cortex cells. Additionally, Si contributed to the widening of metaxylem vessels and pith thickness. These adaptations could restrict Cd translocation to the shoots, thus improving overall growth performance in pea plants. Si was also found to boost the activity of antioxidant enzymes and alleviate oxidative damage. This relatively cheap method of crop protection may bring crop yield improvement in contaminated areas.

In the study of Tan et al. on pakchoi (*Brassica chinensis*), an interesting relationship was elucidated between chloroplast biogenesis (indirectly related to photosynthetic efficiency), ascorbate-glutathione (AsA-GSH) cycle and dose of lead (Pb) present in the soil. Increasing Pb concentrations caused a progressive reduction of the activity of enzymes involved in the AsA-GSH cycle, as well as a decrease in the content of reduced forms of ascorbic acid and glutathione. These changes contributed to the inhibited expression of genes involved in chloroplast development and were manifested in the disturbed structure of chloroplasts, including membranes and stromal sheets, particularly under higher Pb concentrations. These findings broaden current knowledge on the interconnection between photosynthetic apparatus and antioxidant machinery under Pb toxicity.

In summary, the published articles in our Research Topic are in accordance with Paracelsus’s statement *‘Omnia sunt venena, nihil est sine veneno. Sola dosis facit venenum’*, and indicate dose- and time-dependent effects of metallic trace elements on plants. They represent valuable contributions to deciphering plant ability to repair metal-induced damage and describing the networks that make the life of stressed plants easier. Although the tested variety of both metals and priming agents, the general conclusion of all research clearly highlights the crucial role of antioxidant machinery in plant survival under metal exposure. We hope that the results of the presented experiments will be an inspiration for further research in this field, and will facilitate the development of novel solutions against the progressive heavy metal contamination of the environment.

## Author contributions

EM and AW wrote the manuscript. All authors revised the manuscript and gave final approval for publication.
